# Kinetic-Thermodynamic Promotion Engineering toward High-Density Hierarchical and Zn-Doping Activity-Enhancing ZnNiO@CF for High-Capacity Desalination

**DOI:** 10.1007/s40820-024-01371-y

**Published:** 2024-03-04

**Authors:** Jie Ma, Siyang Xing, Yabo Wang, Jinhu Yang, Fei Yu

**Affiliations:** 1 College of Marine Ecology and Environment, Shanghai Ocean University, 201306 Shanghai, People’s Republic of China; 2 School of Civil Engineering, Kashi University, 844000 Kashi, People’s Republic of China; 3 Research Center for Environmental Functional Materials, College of Environmental Science and Engineering, Tongji University, 1239 Siping Road, 200092 Shanghai, People’s Republic of China; 4 School of Chemical Science and Engineering, Tongji University, 1239 Siping Road, 200092 Shanghai, People’s Republic of China; 5 Department of Energy, Environmental & Chemical Engineering, Washington University in St. Louis, St. Louis, MO 63130 USA

**Keywords:** Zinc–nickel metal oxide, High-density hierarchical, Capacitive deionization, Zinc-doping

## Abstract

Despite the promising potential of transition metal oxides (TMOs) as capacitive deionization (CDI) electrodes, the actual capacity of TMOs electrodes for sodium storage is significantly lower than the theoretical capacity, posing a major obstacle. Herein, we prepared the kinetically favorable Zn_x_Ni_1 − x_O electrode in situ growth on carbon felt (Zn_x_Ni_1 − x_O@CF) through constraining the rate of OH^−^ generation in the hydrothermal method. Zn_x_Ni_1 − x_O@CF exhibited a high-density hierarchical nanosheet structure with three-dimensional open pores, benefitting the ion transport/electron transfer. And tuning the moderate amount of redox-inert Zn-doping can enhance surface electroactive sites, actual activity of redox-active Ni species, and lower adsorption energy, promoting the adsorption kinetic and thermodynamic of the Zn_0.2_Ni_0.8_O@CF. Benefitting from the kinetic-thermodynamic facilitation mechanism, Zn_0.2_Ni_0.8_O@CF achieved ultrahigh desalination capacity (128.9 mg_NaCl_ g^−1^), ultra-low energy consumption (0.164 kW h kg_NaCl_^−1^), high salt removal rate (1.21 mg_NaCl_ g^−1^ min^−1^), and good cyclability. The thermodynamic facilitation and Na^+^ intercalation mechanism of Zn_0.2_Ni_0.8_O@CF are identified by the density functional theory calculations and electrochemical quartz crystal microbalance with dissipation monitoring, respectively. This research provides new insights into controlling electrochemically favorable morphology and demonstrates that Zn-doping, which is redox-inert, is essential for enhancing the electrochemical performance of CDI electrodes.

**Supplementary Information:**

The online version contains supplementary material available at 10.1007/s40820-024-01371-y.

## Highlights

- Through facial basicity adjustment, kinetically favorable Zn_x_Ni_1-x_O@CF electrode was formed with a high density hierarchical structure and three dimensional open pores.
- The optimal Zn-doping ratio in Zn_x_Ni_1-x_O@CF has excellent sodium storage and desalination performance (128.9 mg g^-1^).
- The mechanism of Na^+^ intercalation process was studied by electrochemical quartz crystal microbalance with dissipation monitoring in situ test and the activation mechanism of redox-inert Zn-doping on electrode materials was reported.

**Supplementary Information:**

The online version contains supplementary material available at 10.1007/s40820-024-01371-y.

## Graphical abstract

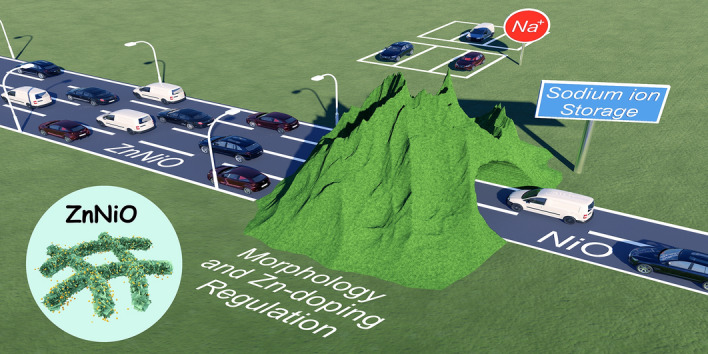

## Introduction

Due to its ease of operation, reduced energy cost, and high work efficiency in comparison with traditional desalination technologies, the capacitive deionization (CDI) technology offers considerable potential to address the severe global water shortage problem [1]. Ion-capture mechanisms in CDI are generally similar to those in sodium-ion batteries (SIBs). Consequently, considerable effort has been devoted to exploring the potential of SIBs as cathode materials for CDI processes [2, 3]. Carbonaceous materials, via the electrical double layer (EDL) mechanism [4–6], had hit a roadblock due to their low adsorption capacity (usually < 25 mg g^−1^) and potential for side reactions [7]. The non-carbon electrode materials mainly included transition metal oxides (TMOs) [8–13], Prussian blue analogs [14–16], polyanionic phosphates [17, 18], MXene [19–21], and layered double hydroxide (LDH) [22, 23], and can achieve high capacity desalination by redox reaction [24]. Among these materials, TMOs comprising the advantages of easy preparation, element diversity, facile morphology control, excellent reversible intercalation pseudocapacity and promising sodium storage theoretical capacitance, have shown great potential for application in CDI [11, 12, 25–33].

However, TMOs still suffers from issues with stacking and poor electrical conductivity, which limit their sodium ion intercalation capacity to a certain extent. In addition, the large size of sodium ions leads to the slow sodiation/desodiation reaction kinetics, resulting in a significantly lower capacity for sodium storage than theoretically possible [28, 29, 32–35]. For example, in the present study [26, 27, 36, 37], even the state-of-the-art electrodes of NiO can only reach about 75% of the theoretical capacity, 2573 F g^−1^ within 0.5 V [38], severely impeding their application [39]. Substantial efforts have been undertaken to enhance the reaction kinetics by the creation of various nanostructures to boost electron conduction and reduce the ion diffusion pathway, thereby reducing energy consumption and increasing adsorption rates [40, 41]. As well, it has been argued that by decreasing the electrode size to a certain nanoscale level, the battery-type electrode material will behave similarly to a capacitive electrode [42, 43]. which made it had battery-level storage capacity combined with both cycle life and adsorption rate. For instance, NiCo_2_O_4_@NiCo-LDH can form different nanostructures depending on the hydrothermal synthesis time, where the optimal nanostructure exhibits a capacitance of up to 5810 mF cm^−2^ [44]. It was also discovered that the ion conductivity of Li_10_GeP_2_S_12_ increases as the particle volume decreases when using advanced nanoscale modeling techniques [45].

Despite the advances made, the actual capacity was still far from the theoretical one, even at an ultralow rate, suggesting that there may be thermodynamic difficulties in sodium storage in addition to the electrochemical kinetics limitations. It was possible to promote sodium intercalation both kinetically and thermodynamically by transition metal doping [46–48]. Transition-metal doping essentially reduced the sodium intercalation energies, rearranged electron distribution and enabled a complete adsorption reaction [39]. Among the promising and hot candidates for electrochemical energy storage and conversion, zinc (Zn) has been widely studied. The doping of Zn in electrode materials had been reported to typically exhibit specific characteristics such as increased electronic conductivity, reaction activity, and surface roughness, resulting in improved electrochemical properties [49–51]. The incorporation of Zn into the ZnNiCo-P can boost charge transfer and enhance ion adsorption processes, considerably improving the electrochemical performance [51]. However, over-doping of Zn may lead to the formation of by-products, as well as instability in the crystallization of the material, resulting in partial leaching/dissolution of the material [11, 49]. Based on the above consideration, it was highly promising for advanced CDI cathodes to find a simple method to adjust the microscopic morphology of electrodes and construct chemically stable electrodes with dual kinetic-thermodynamic enhancement by an appropriate amount of Zn-doping.

Herein, a redox-inert Zn-doping activated high-density hierarchical Zn_0.2_Ni_0.8_O@CF electrode was conveniently synthesized via a one-step hydrothermal method. Simple adjustment of the basicity of the hydrothermal conditions could constrain the rate of OH^−^ production and thus the number of lamellar nuclei formation, giving this electrode a kinetically favorable nanostructure, i.e., increased ion-accessible surface sites and a high-speed ion conduction network. Zn_0.2_Ni_0.8_O electrode demonstrated exceptional CDI performance with outstanding desalination capacity of 128.9 mg_NaCl_ g^−1^, excellent cycling stability and ultra-low energy consumption, which exceeding the desalination capacity of other state-of-the-art CDI electrodes at similar conditions. The density functional theory (DFT) and ex situ XPS analysis showed that Zn doping could not only enhance the electron transfer kinetics through improved conductivity but lower the Na^+^ adsorption energy and improve the adsorption thermodynamics, through enhancing the real activity of surface electroactive sites and redox-active Ni species. Electrochemical quartz crystal microbalance with dissipation monitoring (EQCM-D) revealed the mechanism and high reversibility of Na^+^ intercalation. This work provided a new perspective on a simple way to regulate electrochemically favorable nanomorphology and the critical role of redox-inert Zn-doping as an active promoter for advanced CDI electorate design.

## Experimental

### Materials Preparation

A piece of 3 cm × 3 cm × 0.1 cm carbon felt was immersed in a mixture of H_2_SO_4_ (98%) and HNO_3_ (68%) at a volume ratio of 1:3 and placed in a water bath at 80 °C for 3 h. The carbon felt was then removed from the mixture and rinsed with deionized water to a neutral pH and get the prefabricated carbon felt.

To prepare Zn_x_Ni_1 − x_(OH)_2_ carbon felt electrode, 6 g of urea, 3.7 g of NH_4_F, 14.75 g of Ni(NO_3_)_2_·6H_2_O, and 1.48 g of Zn(NO_3_)_2_·6H_2_O were dissolved in a volume of 500 mL of deionized water at room temperature in to obtain mixture A. The pH of the pre-solution was adjusted to 4. The hydration reactor contained pCF and pre-solution A was sealed at a constant temperature (140 °C) for 2 h to obtain a prefabricated carbon felt electrodes with surface loading of Zn_x_Ni_1 − x_(OH)_2_. The dried Zn_x_Ni_1 − x_(OH)_2_ was placed in a tube furnace with nitrogen gas environment to obtain a Zn_x_Ni_1 − x_O electrode. More details are in Sect. S3 of Supporting Information.

### Electrochemical Test

The Zn_x_Ni_1 − x_O were directly used as working electrode. An electrochemical station (CHI660D, Chenhua Instruments Co.) was used for all electrochemical tests. CV was swept between − 0.4 and 0.8 V under certain scan rates (1–60 mV s^–1^), and GCD was measured at the uniform voltage window with various specific currents (1–6 mA cm^–2^). Cyclic voltammetry (CV), galvanostatic charging/discharging (GCD), and electrochemical impedance spectroscopy (EIS) tests were conducted on a three-electrode system consisting of a working electrode, Pt (counter electrode), and Ag/AgCl (reference electrode) in 1 M NaCl. More details are in Sect. S3 of Supporting Information.

### Desalination Experiments

The flow-by CDI stack is composed of glass plates, silica gel gaskets, a cation/anion exchange membrane (CEM/AEM), and a chamber with dimensions of 0.8 cm × 4 cm × 4 cm. An activated carbon electrode served as the anode, and a Zn_x_Ni_1 − x_O electrode served as the cathode. All desalination experiments were carried out under a constant current with a fixed flow rate, a fixed initial NaCl concentration and a fixed volume, while other operational parameters, including the different activated electrodes, current density, were varied to obtain the best desalination capacity. More details are in Section S4.

## Results and Discussion

### Material Characterization of Zn_x_Ni_1 − x_O@CF

**Fig. 1:**
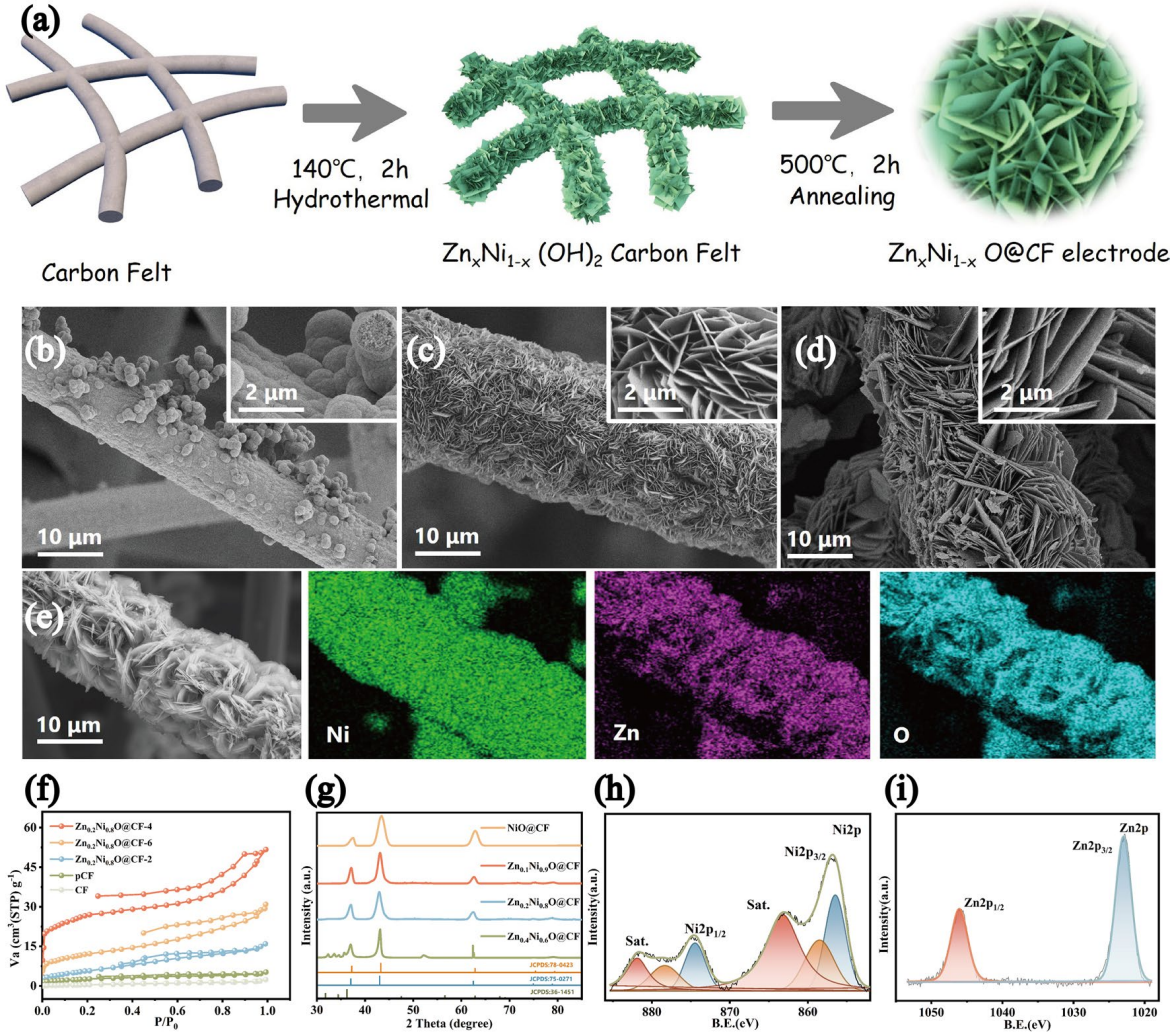
**a** Schematic illustration of the layer Zn_x_Ni_1 − x_O@CF preparation. SEM of the **b** Zn_0.2_Ni_0.8_O@CF-2, **c** Zn_0.2_Ni_0.8_O@CF-4 and** d** Zn_0.2_Ni_0.8_O@CF-6. **e** EDS mapping image of Zn_0.2_Ni_0.8_O@CF-4, **f** N_2_ adsorption/desorption isotherms and **g** XRD patterns of Zn_x_Ni_1 − x_O@CF with different Zn-doping. XPS spectrum of Ni 2*p* **h** and Zn 2*p* **i** for Zn_0.2_Ni_0.8_O@CF

Control the elemental ratio of Zn–Ni dosage of 1:4, a one-step hydrothermal method was used to synthesize Zn_0.2_Ni_0.8_(OH)_2_ nanosheets in situ on the surface of prefabricated CF and we obtained Zn_0.2_Ni_0.8_O@CF electrodes by annealing at 500 °C (Fig. 1a). Samples obtained based on pre-mixtures of different basicity (pH = 2, 4, and 6), named Zn_0.2_Ni_0.8_O@CF-2, Zn_0.2_Ni_0.8_O@CF-4, and Zn_0.2_Ni_0.8_O@CF-6, respectively (more details in Supporting Information). Distinctive from routine methods, we found that alterations in the basicity of the hydrothermal conditions may have a considerable effect on electrode morphology (Fig. 1b–d). Zn_0.2_Ni_0.8_O@CF-4 nanosheets with diameter of about 500 ~ 1500 nm and thickness of 30 ~ 60 nm would be formed in situ on CF in a high-density hierarchical structure. The Zn_0.2_Ni_0.8_O@CF-4 had a loosely packed morphology, but the nanosheets were strongly connected, which increased electrolyte permeability and formed the improved electron pathways by the formation of three-dimensional networks. This resulted in a faster sodiation adsorption kinetics and an enhanced pseudocapacitance [52]. Base dissociation rates determined the reaction kinetics between the metal precursor counterions and dissociated OH^−^, resulting in different morphologies [53]. However, when the pH of the pre-solution is 2, the surface of CF formed micron porous spheres without the formation of sheet structure; when the pH = 6, the sheets were larger and thicker, with more serious stacking phenomenon. On the basis of this assumption, by regulating the pH of the pre-solution to regulate basicity of hydrothermal condition, a high-density hierarchical layered Zn_0.2_Ni_0.8_O@CF would be produced in an energetically favorable state (Fig. 1c) and possess the best electrochemical properties, which could be explained by Ostwald ripening [54, 55]. As shown in Fig. 1e, the results of energy dispersive spectrometer (EDS) mapping showed that Zn, Ni, and O elements were evenly spread across the surface of carbon felts, demonstrating the successful synthesis of Zn_0.2_Ni_0.8_O@CF; in addition, C cannot be observed almost in the region where the nanosheets were attached, also indicating the synthesis of a high-density Zn_0.2_Ni_0.8_O@CF layer structure on the carbon fiber surface.

Figures 1f and S1 display the specific surface area (SSA) and corresponding pore size distribution (PSD) results. Zn_0.2_Ni_0.8_O@CF-4 exhibited a type IV isotherm and had the largest SSA (94.341 m^2^ g^−1^, Table S1), which was the signature of hierarchical micro-mesoporous structure and consistent with the BJH pore size fitting results. Microporous structure could enhance the desalination capacity by offering multiple adsorption sites for ion accommodation; while the mesopores facilitated ion transport due to their wide pore openings and thus enhanced the desalination rate [56, 57]. In contrast, other simples exhibited the type III N_2_-sorption isotherm indicating there were no pores or a little microporous. This was because, under more reasonable hydrothermal conditions, the Zn_0.2_Ni_0.8_O@CF sheet structure was more homogeneous and less self-stacking (compared to Fig. 1d), which eventually formed a rich pore structure and fast ion transport pathways. In this case, the pre-solution preparation condition of pH = 4 was chosen in all later experiments. Therefore, the material was named as Zn_0.2_Ni_0.8_O@CF.

The XRD of prepared Zn_0.2_Ni_0.8_O@CF in different pH conditions (Fig. S2) all matched well with the Zn_0.2_Ni_0.8_O@CF standard spectrum (JCPDS No. 75-0271), and combined with the absence of peaks of ZnO and the presence of Zn element in the electrode (Fig. 1e), suggested that Zn was doped in the Zn_0.2_Ni_0.8_O@CF crystalline phase rather than just physically blending two unary phases. The higher peak strength of the Zn_0.2_Ni_0.8_O@CF materials was also attributed to the Zn-doping, indicating a higher degree of crystallinity. However, this conclusion did not apply in all cases. We also adjusted the dosing ratios of Zn and Ni and named them as NiO@CF, Zn_0.1_Ni_0.9_O@CF and Zn_0.4_Ni_0.6_O@CF, respectively. As shown in Fig. 1g, when Zn was added in a certain range, Zn was mainly doped in the crystalline phase of NiO: Zn_0.1_Ni_0.9_O@CF and Zn_0.2_Ni_0.8_O@CF exhibited XRD patterns similar to NiO (JCPDS No. 78-0423); but when the proportion of Zn-doping increased (such as Zn_0.4_Ni_0.6_O@CF), the crystalline phase of ZnO appears (JCPDS No. 36-1451), destabilizing the crystal and making it easier to decompose [11], resulting in less electrochemical stability for the electrodes. Jia et al. [46] also found that the ratio of Zn-doping was essential for improving the electrochemical performance. Therefore, to further investigate the effect of Zn-doping amount, different Zn-doping ratios on the electrode performance were mainly discussed in the subsequent investigations.

The chemical states of different Zn_x_Ni_1 − x_O@CF simples were investigated via XPS (Fig. S3a). Two spin–orbit splitting peaks with a spin-energy separation of 17.6 eV were observed in the high-resolution spectra of Ni 2*p* (Fig. 1h) [58–60]. The Zn 2*p* spectra in Fig. 1i showed the Zn 2*p*_1/2_ and Zn 2*p*_3/2_ peaks of the Zn 2*p* doublet at approximately 1045.87 and 1022.89 eV, respectively [61]. It also provided the exact formulas of Zn_x_Ni_1 − x_O, which was Zn_0.12_Ni_0.88_O, Zn_0.18_Ni_0.82_O, and Zn_0.31_Ni_0.69_O and closed to the theoretical ratio. O 1*s* (Fig. S3b) could be decomposed into hydroxyl groups at the surface, O^2−^ ions in the crystal structure, and carbon dioxide or adsorbed water molecules. The presence of interfacial OH^−^ contributed to the hydrophilicity of Zn_x_Ni_1 − x_O@CF, which was confirmed by the ultralow water contact angle (Fig. S4). Additionally, the hydration shells or crystallographic water formed in metal oxides could speed up the ion transport [62].

### Electrochemical Properties

**Fig. 2:**
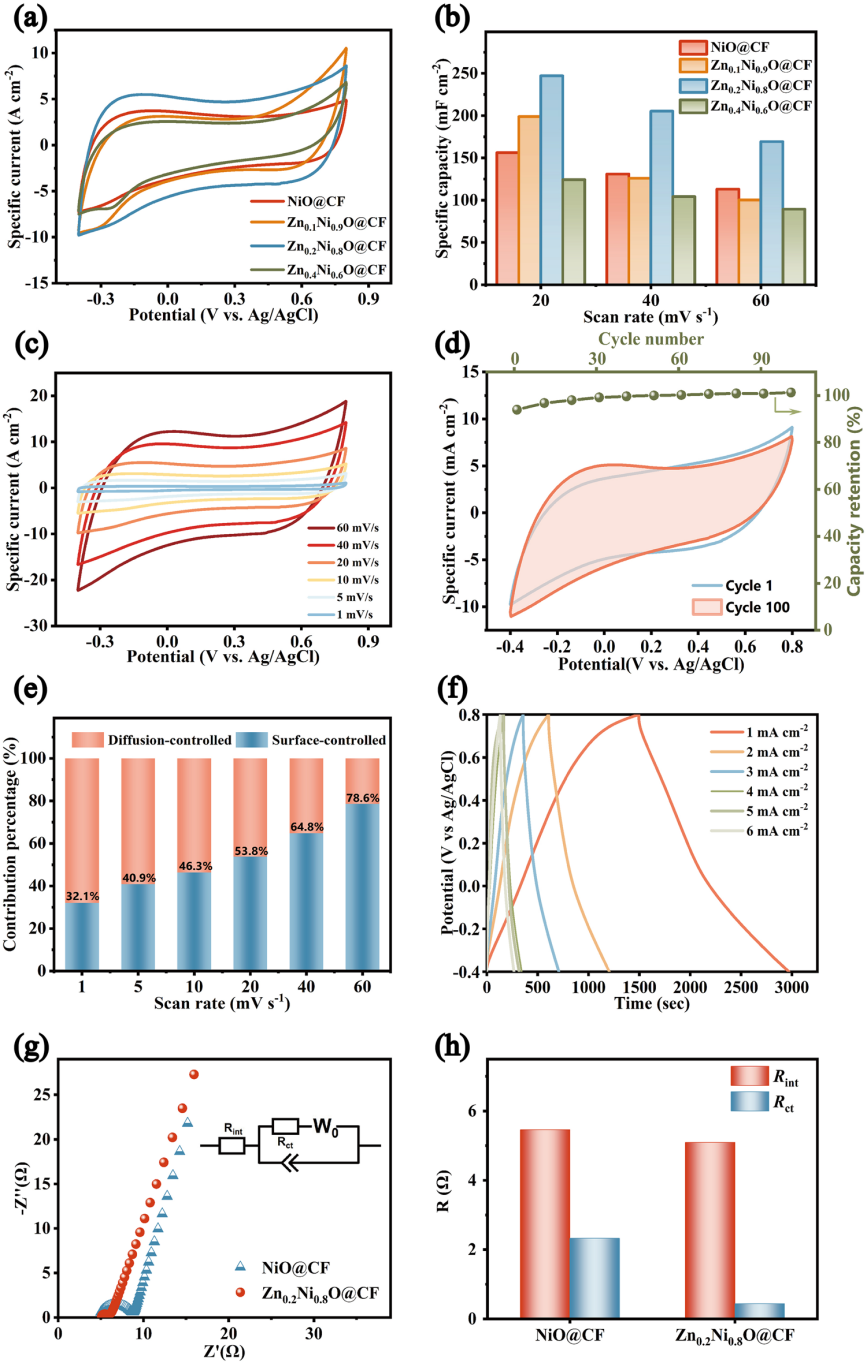
**a** CV curves of NiO@CF, Zn_0.1_Ni_0.9_O@CF, Zn_0.2_Ni_0.8_O@CF and Zn_0.4_Ni_0.6_O@CF and **b** specific capacity of various scan rates. **c** CV curves of Zn_0.2_Ni_0.8_O@CF measured at different scan rates and **d** after 100 cycles at 10 mV s^−1^. **e** Normalized contribution ratios of surface-/diffusion-controlled capacities and **f** GCD profiles of Zn_0.2_Ni_0.8_O@CF.** g** Nyquist plots and **h** simulated internal and charge transfer resistance of NiO@CF and Zn_0.2_Ni_0.8_O@CF

Electrochemical tests were performed in a 1 mol L^−1^ NaCl solution using a three-electrode system to further investigate the Zn_x_Ni_1 − x_O@CF electrode performance and the effect of different Zn-doping amounts. CV curves of the electrodes presented a quasi-rectangular shape without prominent redox peaks (Fig. 2a), indicating that the sodium storage mechanism could be attributed to pseudocapacitance [63]. Regardless of the sweep speeds tested, Zn_0.2_Ni_0.8_O@CF consistently exhibited the highest area-specific capacity among the four materials (Figs. 2b and S5a) and the best electrochemical performance (414.647 mF cm^−2^ at 1 mV s^−1^), and its capacity reached 247.09 mF cm^−2^ at 20 mV s^−1^, 1.25 times that of Zn_0.1_Ni_0.9_O@CF and 1.59 times that of NiO@CF. Considering the redox-inert effect of the Zn in the pseudocapacitance storage [46, 51], the specific capacity of Zn_0.2_Ni_0.8_O@CF electrode (18% redox-inert Zn/82% redox-active Ni) was even slightly superior to the NiO@CF electrode (100% redox-active Ni), indicating Zn-doping of the Zn_0.2_Ni_0.8_O@CF material could promote activity through underlying mechanisms. The poor performance of Zn_0.4_Ni_0.6_O@CF electrode may be attributed to the ZnO phase formation due to the redundant zinc salt (Fig. 1i), resulting in a blend of ZnO and Zn_x_Ni_1 − x_O.

Therefore, the Zn: Ni ratio of the pre-mixture solution was set to 1:4. The CV curves in Fig. 2c showed similar trends at different scan rates exhibited excellent capacitive reversibility of Zn_0.2_Ni_0.8_O@CF, which was also characterized by repeated CV cycling and displayed no significant change after 100 cycles at 10 mV s^−1^ (Fig. 2d). The high-level capacity retention was also related to the high homogeneity of the hierarchical interconnected nanosheet network, effectively mitigating the bulk phase expansion of ion intercalation [64, 65].

In addition, Fig. 2e explores the current contribution of the surface- and diffusion-control process of the Zn_0.2_Ni_0.8_O@CF electrode. As shown in Figs. 2e and S5b, c, the Zn_0.2_Ni_0.8_O@CF electrode showed a dominant capacitive contribution, reaching a maximum of 78.6% at 60 mV s^−1^. It has been suggested that battery-like electrode materials can exhibit capacitive behavior when their size is reduced to a certain nanoscale level (typically less than 10 nm) [43], which may explain the high pseudocapacitance of the Zn_0.2_Ni_0.8_O@CF electrode, likely due to the kinetic promotion of the redox reaction on the surface of the ultrathin nanosheets. Besides, no plateau occurred in galvanostatic charge–discharge (GCD) profiles (Fig. 2f), which further demonstrated the pseudocapacitance behavior and indicated that adsorption reactions were occurring at the surface rather than in the bulk. These may be attributed to the low degree of stacking of the nanosheets obtained by morphological modulation with a highly open structure and short diffusion pathways, which enabled the electrodes to possess a high number of active sites and faster reaction kinetics [52, 66, 67]. Above all, the Na^+^ removal process was shown by Eqs. (1) and (2):

1 $${\text{Zn}}_{0.2}{\text{Ni}}_{0.8}O+x{\text{Na}}^{+}+x{\text{e}}^{-}\rightarrow {\text{Na}}_{x}{\text{Zn}}_{0.2}{\text{Ni}}_{0.8}O$$

2 $${\text{Zn}}_{0.2}{\text{Ni}}_{0.8}O{({\text{OH}})}_{n}^{-}+n{{\text{Na}}}^{+}\rightarrow{\text{Zn}}_{0.2}{\text{Ni}}_{0.8}O{({\text{OH}})}_{n}{\text{Na}}_{x}$$

To further investigate the effectiveness of Zn-doping, electrochemical impedance measurements (EIS) were measured (Fig. 2g). Parameters fitted values were shown in Fig. 2h and Table S2. Zn_0.2_Ni_0.8_O@CF electrode showed lower internal resistance (*R*_int_) value (5.10 Ω) compared to NiO@CF (5.46 Ω), indicating a lower proportion of internal resistance to charge consumption and higher charge efficiency. The charge transfer resistance (*R*_ct_) value of the Zn_0.2_Ni_0.8_O@CF electrode (0.44 Ω) was also lower than NiO@CF (2.33 Ω), which meant Zn_0.2_Ni_0.8_O@CF had a better conductivity and superior electrochemical kinetics [17].

### CDI Performance

**Fig. 3:**
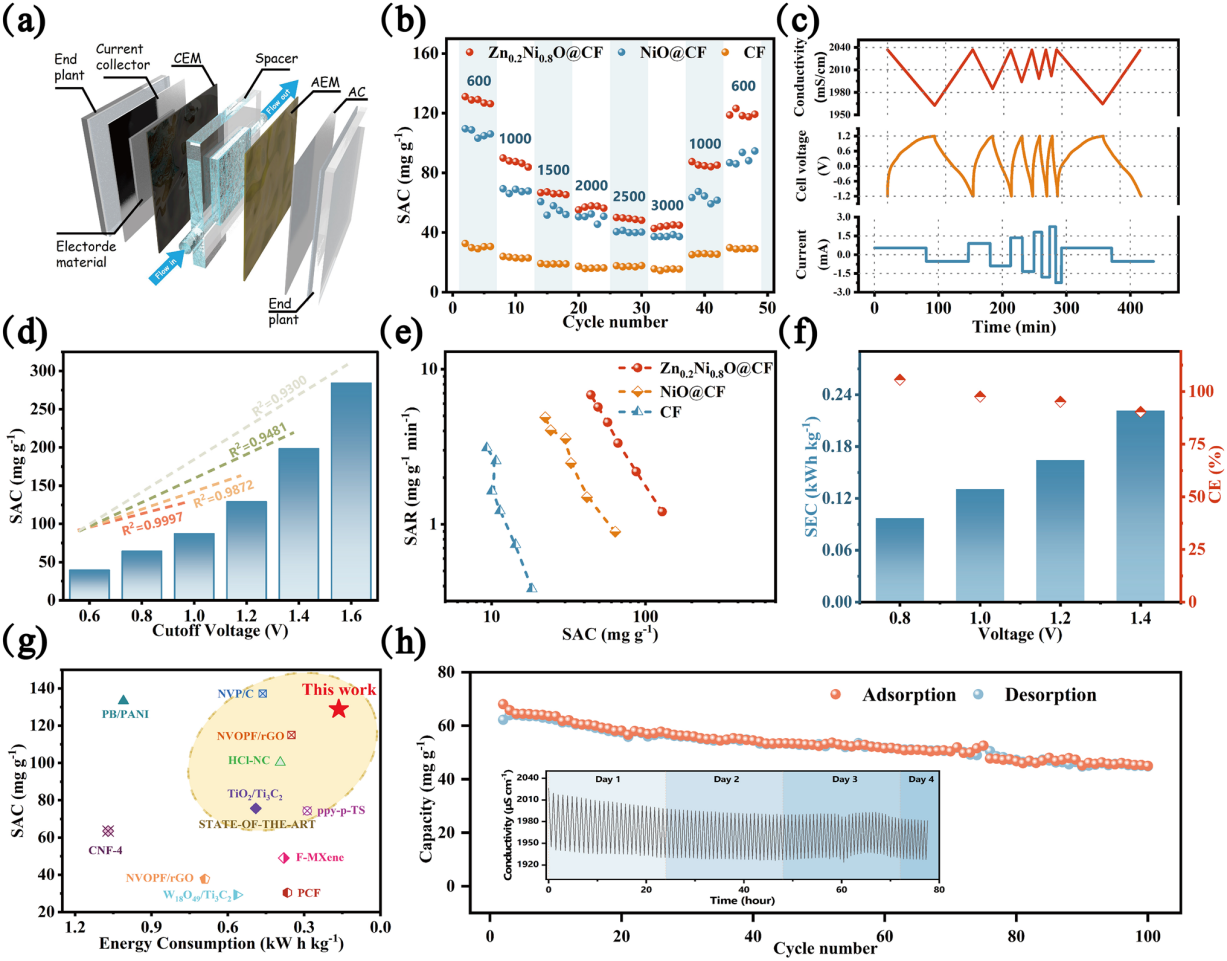
**a** Schematic diagram of CDI process. **b** SAC of Zn_0.2_Ni_0.8_O, NiO and CF electrodes at different current densities. **c** The profiles of the conductivity, voltage, and current at various specific current. **d** SAC of Zn_0.2_Ni_0.8_O at different cut-off voltages. **e** Ragone plots of various electrodes.** f** CE and SEC of Zn_0.2_Ni_0.8_O. **g** Comparison of SAC and SEC between Zn_0.2_Ni_0.8_O and other state-of-the-art materials. **h** Cycling and regeneration performance of Zn_0.2_Ni_0.8_O at 1500 mA m^−2^ over 100 cycles. Inset is the real-time conductivity and voltage profiles

Activated carbon (AC) and the prepared materials were used as the cathode and the anode in CDI system with 1 g L^−1^ of NaCl, respectively (Fig. 3a). In constant current operation mode, specific current was a critical ingredient for engineering applications, as it was closely linked to energy consumption and desalination efficiency. At the specific current of 600 mA m^−2^, specific adsorption capacity (SAC) of the Zn_0.2_Ni_0.8_O@CF electrode was as high as 128.9 ± 1.9 mg g^−1^, which was the highest among all materials. Zn_0.2_Ni_0.8_O@CF electrode (18% redox-inert Zn/82% redox-active Ni) had a slightly higher SAC than the NiO electrode (100% redox-active Ni), also revealing the underlying activity-promoting mechanisms of Zn-doping.

As the current density returned to original level, the retention rate of Zn_0.2_Ni_0.8_O@CF electrode of SAC was 93.06% from Fig. 3b, and superior to the NiO electrode (86.91%), indicating that with appropriate Zn-doping, the crystal structure of Zn_0.2_Ni_0.8_O@CF remained relatively intact, and it had a high reversible capacity, which was also consistent with the analysis of Zn_0.2_Ni_0.8_O@CF crystallinity in XRD. SAC arising from current collector CF was negligible, thus the main capacity contribution came from Zn_0.2_Ni_0.8_O material. Besides, a higher specific current led to a faster charge transfer (Fig. 3c), with a greater rate of electron transfer observed per unit time, resulting in a higher salt adsorption rate (SAR) [15, 68]. SAR was 6.81 ± 0.19 mg g^−1^ min^−1^ at 3000 mA m^−2^, nearly six times greater than the rate at 600 mA m^−2^ (Fig. S6a) and practically one of the highest SAR materials available today (Table S3). The cut-off voltage, which marked the point of potential reversal, has a significant impact on the NaCl removal capacity. When the voltage interval expanded from − 0.6/ + 0.6 to − 1.4/ + 1.4 V, the desalination capacity of Zn_0.2_Ni_0.8_O@CF increased from 39.44 ± 0.18 to 198.3 ± 1.9 mg g^−1^ (Fig. 3d). Wider voltage intervals correspond to longer charging times. Consequently, a greater amount of charge was stored at the electrode, allowing it to take part in the desalination process. As shown in the CDI Ragone plot (Fig. 3e) of all prepared electrodes, it was clearly observed that the Zn_0.2_Ni_0.8_O@CF displays the highest SAR, SAC and better desalination performance. In addition, a comparison with samples synthesized at different pH conditions with different doping amounts was shown in Fig. S6b and also indicated that Zn_0.2_Ni_0.8_O@CF had the best desalination performance among all samples (Zn_0.4_Ni_0.6_O@CF had severe leaching due to crystal instability and the presence of ZnO [49], and the conductivity continued to rise, so the desalination capacity was not calculated). These results convincingly verified the excellent desalination performance of the Zn_0.2_Ni_0.8_O@CF electrode and the underlying electrochemical enhancement mechanism of the Zn-doping.

A crucial metric for CDI performance was energy consumption [2]. When the voltage range was ± 1.2 V, the SEC of Zn_0.2_Ni_0.8_O@CF was 0.164 kW h kg^−1^ of NaCl, as shown in Fig. 3f, which increased to 0.222 kW h kg^−1^ of NaCl when the voltage interval was extended to ± 1.6 V, approaching the lower limit of constant-current CDI devices when tested in similar conditions [15, 56, 69]. The higher coulombic efficiency (CE) was close to 100% at all cut-off voltages, also leading to lower SEC. Accordingly, the energy recovery ratio was maintained at a stable value (28.6% ~ 23.6%) and was among the highest of the current cutting-edge electrodes (the recyclable energy was donated as *E*_a_ and *E*_c_ in Fig. S7), deriving from a lower ion diffusion energy barrier and promoted kinetics reaction [70] due to appropriate morphology control and Zn-doping. Unlike the commonly used Ragone diagram (known as Kin–Yoon diagram, SAR vs. SAC), we underlined the significance of energy consumption. SEC was a crucial metric in engineering practice due to the global energy crisis and the economic benefits of desalination. Compared with recently reported CDI electrodes (including carbonaceous and faradaic materials) (Fig. 3g) [15, 17, 18, 56, 70–77], it was clearly observed that Zn_0.2_Ni_0.8_O@CF displayed the highest SAC and lowest SEC among the cutting-edge CDI electrodes. The excellent CDI performance of Zn_0.2_Ni_0.8_O@CF originated from kinetic promotion through a hierarchical nanosheet interconnection network structure with high active sites, enhanced intrinsic electron transfer and adsorbed activation of Zn-doping. Therefore, Zn_0.2_Ni_0.8_O@CF has the potential to be put into actual practice.

To further verify the long-term cycling performance of Zn_0.2_Ni_0.8_O@CF electrodes, 100 desalination cycles were carried out for about four days. The SAC of Zn_0.2_Ni_0.8_O@CF showed excellent reversibility of 86.2% (Fig. 3h), proving no signs of significant performance decay. Furthermore, the adsorption and desorption capacity remained nearly constant, indicating that the system was in a state of dynamic equilibrium (inset of Fig. 3h). Moreover, the morphology of Zn_0.2_Ni_0.8_O@CF nanosheets remained largely unchanged, except for slight aggregation, after 100 cycles (Fig. S8). In comparison to other carbon–metal composite electrodes, Zn_0.2_Ni_0.8_O@CF showed both a high capacity and excellent stability. The outstanding long-term performance demonstrated that Zn_0.2_Ni_0.8_O@CF would be a promising electrode for CDI application.

### Desalination Mechanism

**Fig. 4:**
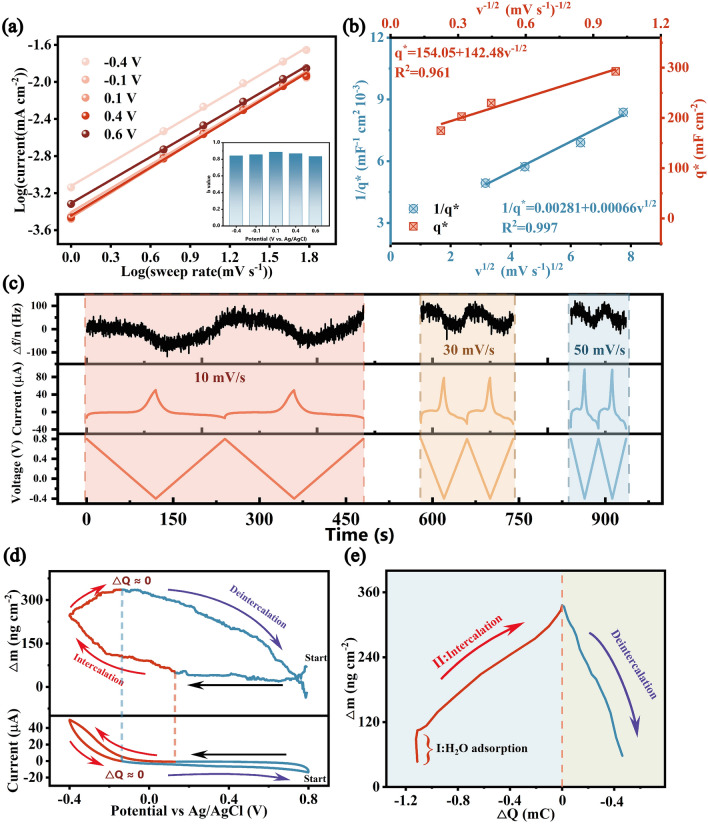
**a** Calculation of b-values of Zn_0.2_Ni_0.8_O based on CV curves. **b** The relationship between 1/*q*^∗^ and v^1/2^ and between *q*^∗^ and v^−1/2^. **c** △f/5 responses of Zn_0.2_Ni_0.8_O from EQCM-D during CV at different scan rates. The simultaneous **d** mass change and current response and **e** change in electrode mass versus charge passed during Zn_0.2_Ni_0.8_O electrode adsorption/desorption processes at 10 mV s^−1^ between − 0.4 ~ 0.8 V. Blue region highlights the process of adsorption and yellow highlights the process of desorption

To further investigate the ion removal and charge storage kinetics, through the CV curves, we illustrated the power-law relationship. The current followed the law of diffusion-controlled if the *b*-value is about 0.5, which is usually seen in battery-like systems, while a value of 1.0 indicated an ideal surface-controlled case [23]. According to Fig. 4a, the calculated *b*-values were higher than 0.8 for all voltages. Such a high *b*-value was consistent with strong pseudocapacitive nature of Zn_0.2_Ni_0.8_O@CF electrode, thus providing superior rate capability and lower energy consumption [78]. The Trasatti analysis method was used to further analyze electrochemical kinetics of the Zn_0.2_Ni_0.8_O@CF sample. This approach differentiated the surface-controlled capacity of Zn_0.2_Ni_0.8_O@CF electrode into "inner" and "outer" surface control (more details in Supporting Information) [63]. The "inner" surface referred to the regions of difficult accessibility, while the "outer" surface mainly came from the surface exposed directly to ions and was unaffected by sweep rates. The calculation was based on Eqs. (3) and (4):

3 $$\begin{array}{*{20}c} {q^{*} = q_{{\text{s,out}}} + A_{1} \nu^{ - 1/2} (\nu \to \infty )} \\ \end{array}$$

4 $$\begin{array}{*{20}c} {q^{{{*} - 1}} = q_{s}^{ - 1} + A_{2} \nu^{1/2} (\nu \to 0)} \\ \end{array}$$

where *q* ∗ was the voltammetric charge, *q*_s,out_ was the "outer" capacity, and *q*_s_ was the surface-controlled capacity. *q*_s,out_ was calculated to be 154.1 mF cm^−2^, constituting 43.2% of *q*_s_, which was a relatively very high value [79].

The specific capacitance was 82.13% of the total specific capacitance at the scan rate of 1 mV s^−1^, indicating a prominent electrochemical utilization ratio for Zn_0.2_Ni_0.8_O@CF. This indicated that Zn_0.2_Ni_0.8_O@CF electrode offered rapid, capacitor-like ion removal and charge transfer (Fig. 4b), which was beneficial to enhance charge-storage kinetics.

To gain further insight into the mass transport of Na^+^ during the adsorption process, we detected the relationship between mass and charge capacity change of electrode materials in the electrochemical process by EQCM-D. As shown in Fig. 4c, significant changes in the frequency factor of the Zn_0.2_Ni_0.8_O@CF electrode can be observed during the CV process. Under the condition of various scan rates, the frequency responses basically returned to their initial values and showed good periodic changes, indicating that on the experimental timescale, the mass changes were recoverable and excellent stability of the Zn_0.2_Ni_0.8_O@CF electrode [80]. EQCM-D results of the 3rd cycle showed that the frequency (Δf_5_/5) decreased at varying scan rates (Fig. S9a), indicating that the mass of the Zn_0.2_Ni_0.8_O@CF electrode increased qualitatively as a result of the Na^+^ adsorption process; additionally, Δf_5_/5 returned to approximately 0 Hz, demonstrating the Na^+^ desorption process caused the decrease in the electrode mass. It was noteworthy that dissipation factor (D) corresponding change showed a similar trend, decreasing first and then returning to the initial value (Fig. S9b), indicating the absorption of ions on the Zn_0.2_Ni_0.8_O@CF electrode was reversible, thus confirming the stability of the Zn_0.2_Ni_0.8_O@CF electrode. The decrease in D when the electrode lost energy quickly implied that adsorbate on the Zn_0.2_Ni_0.8_O@CF surface was rigid and compact [81]. In this case, CV curve of the 3th cycle and the simultaneous EQCM-D response were shown in Fig. 4d, and the corresponding △m − △Q plot was calculated and shown in Fig. 4e. We found that the electrode mass increases uniformly when the material charge increases; while when the current was reversed (△Q ≈ 0), the △m trend changes and the electrode mass starts to decrease, indicating that the material has excellent pseudocapacitive properties and the de-/intercalation process has a high sensitivity to the current response due to the rapid transfer process. The mass change of electrode closely followed the theoretical mass change in stages II and III, indicating that all of the charges were applied for sodium-ion intercalation and no other side reactions occurred. The Sauerbrey's equation was applied to analyze the EQCM-D results to quantify the mass changes of the Zn_0.2_Ni_0.8_O@CF [82]. The Red I region of the △m increased without the change in △Q may be the adsorption process of water molecules. The MPE value in the adsorption region (Red II) was 20.68 g mol^−1^, which was marginally lower than 23 g mol^−1^, demonstrating that Na^+^ adsorption was the main process in this region after partial desolvation of sodium hydrate. However, in the desorption region (Blue), the MPE value was 45.16 g mol^−1^, indicating that this process was co-deintercalation of a water molecule and a Na^+^ ion. The EQCM-D data revealed the excellent pseudocapacitive properties of the material, the mechanism of the ion storage process (intercalation of one sodium ion and co-deintercalation of one sodium ion with one water molecule) and a high degree of sodium-ion adsorption reversibility within the electrochemical processes of sodium storage of Zn_0.2_Ni_0.8_O@CF.

It was widely recognized that the electrochemical performance was closely linked to the surface electroactive sites. To comparatively investigate surface chemical structure of Zn_0.2_Ni_0.8_O@CF and NiO@CF electrodes, the ex-situ XPS was then used. Figure S10 shows the Zn 2*p* spectra of Zn_0.2_Ni_0.8_O@CF electrode in pristine states and in full adsorption/desorption states.

**Fig. 5:**
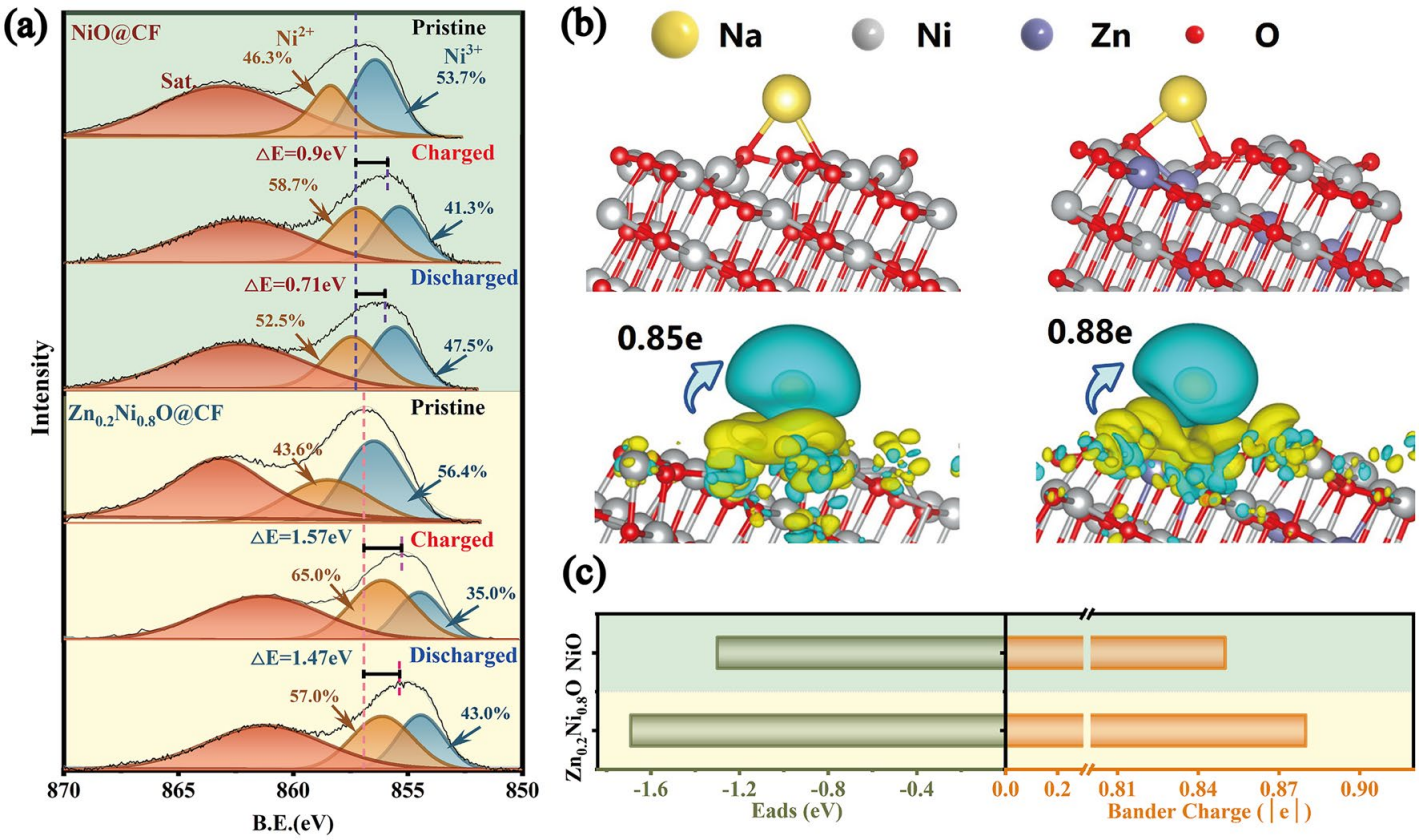
**a** Ex situ XPS spectra/data of Ni 2*p*_3/2_ of NiO and Zn_0.2_Ni_0.8_O electrodes in original states and in completely adsorption/desorption states after the third desalination cycle at ± 1.4 V at 1000 mA cm^−2^. **b** Side view of relaxed adsorption configurations for corresponding *E*_ads_ for Na^+^ on NiO and Zn_0.2_Ni_0.8_O with (225) surfaces, along with the Bader charge and difference charge density analysis. **c** *E*_ads_ and Bader charge for single Na^+^ analysis of the electrodes

It was observed that the Zn valence remained unchanged within the electrochemical processes, which once again proved that Zn was a redox-inert specie in the Zn_0.2_Ni_0.8_O@CF. However, in this case, an interesting finding has been found, that was, the electrochemical performance of Zn_0.2_Ni_0.8_O@CF electrode, which contained 82% redox-active Ni species and 18% Zn redox-inert species, was even slightly better than that of the NiO@CF electrode, which was composed of 100% redox-active Ni species. Thus, an attempt has been made to elucidate the hidden reasons for the interesting phenomenon from the perspective of activity–structure relationship. Figure 5a displayed ex situ XPS spectra of Ni 2*p*_3/2_ of NiO@CF and Zn_0.2_Ni_0.8_O@CF electrodes in original states and in fully adsorption/desorption states at the third desalination cycle of 1000 mA cm^−2^. Upon careful comparison of the changes in peak binding energy between original and completely adsorbed/desorbed states (Table S4), it can be seen that the Zn_0.2_Ni_0.8_O@CF electrode exhibited much larger changes of Ni 2*p*_3/2_ (1.47/1.57 eV) than the NiO@CF electrode (0.9/0.71 eV); furthermore, the relative ratio changes of Ni with 3 + /2 + valences of the Zn_0.2_Ni_0.8_O@CF electrode in fully adsorbed and desorbed states (Ni^3+^ → Ni^2+^: △Ni 8%) are higher than that (Ni^3+^ → Ni^2+^: △Ni 6.2%) of the NiO@CF. The change in the ratios of Ni valences indicated the presence of different electroactive sites on surface during electrochemical processes. The higher conversion between Ni^3+^ and Ni^2+^ species during the adsorption/desorption process suggested more oxidations and reductions of Ni active species (Ni^3+^/Ni^2+^) with larger charge transfer ability [46], proving the superior electroactive sites on surface and promoted reaction kinetics of the Zn_0.2_Ni_0.8_O@CF due to Zn-doping.

To gain better insights of the improved performance of Zn_0.2_Ni_0.8_O@CF electrode due to Zn-doping, we employed DFT to analyze Na^+^ adsorbed thermodynamics, including Na^+^ adsorbed energy and difference charge density in the Zn_0.2_Ni_0.8_O@CF and NiO@CF electrodes. It was reported that the adsorption capability and charge transfer ability of surface-active metals of electrodes were closely linked to electrochemical activity [83, 84]. Figures 5b, c and S11 show Bader charge analysis, difference charge density, the adsorption energy of Na^+^ (*E*_ads_) and the optimized structural models of the NiO@CF and Zn_0.2_Ni_0.8_O@CF with (225) surfaces. In Fig. 5c, the Zn_0.2_Ni_0.8_O@CF showed much more negative average* E*_ads_ of Na^+^ (− 1.96 eV) to those of the NiO (− 1.30 eV), proving the superior thermodynamic adsorption of Na^+^. In all instances, there was a net increase in electrons on the O atoms, while there was a net decrease in electrons on Na^+^, indicating a substantial charge transfer from Na^+^ to surface [85]. The analysis of Bader charge (Fig. 5c) additionally confirmed this result, showing that 0.88e were transferred from Na^+^ to the surface of Zn_0.2_Ni_0.8_O@CF, which is higher than the amount transferred to the NiO@CF electrode (0.85e). Since the neutral and isolated systems were utilized in the system, electrons could not be transferred to external circuit. Consequently, transfer of a greater number of electrons from Na^+^ to the surface implied improved capability of electronic transfer of the surface to the external circuit when the constant voltage was applied. So, in this case, the superior adsorption thermodynamic of Na^+^ and charge transfer capability could promote the electrochemical reactions and consequently improve the reaction activity. The analysis of DFT further illustrated that Zn_0.2_Ni_0.8_O@CF electrode activity can be adequately promoted by the Zn-doping.

## Conclusion

In this study, by simply adjusting the pH of the pre-mixture conditions, we constrained the rate of OH^−^ generation and thus the number of lamellar nuclei formation, then prepared the Zn_0.2_Ni_0.8_O@CF electrode with a high-density hierarchical structure with three-dimensional open pores, which has more ion accessible surface area and high-speed ion conductive network. The Zn_0.2_Ni_0.8_O@CF electrode exhibited high desalination capacity (128.9 mg_NaCl_ g^−1^), fast rate capability (1.21 mg_NaCl_ g^−1^ min^−1^), low energy consumption (0.164 kW h kg_NaCl_^−1^), and high cyclability, outperforming the desalination performance of cutting-edge CDI electrodes. In addition to the kinetic convenience provided by the surface nanostructures, the excellent electrochemical performance was also improved from kinetic and thermodynamic perspectives by appropriate amounts of Zn-doping. Ex situ XPS and DFT analysis demonstrated that Zn-doping can be an active promoter to enhance the surface electroactive sites, real activity of redox-active Ni species, thus reduce the sodium adsorption energy of the Zn_0.2_Ni_0.8_O@CF electrode. The EQCM-D provided unequivocal evidence that the Zn_0.2_Ni_0.8_O@CF had high-speed charge-mass correspondence and Na^+^ was inserted into the material alone, and one Na^+^ ion was co-deintercalation with one water molecule. In this study, a new horizon was opened regarding the regulation of electrochemically favorable micromorphology, and the redox-inert Zn-doping was pivotal to promoting actual activity of reaction sites.

## Supplementary Information

Below is the link to the electronic supplementary material.

Supplementary file1 (PDF 1034 kb)
