# Supplementary material for: Kinetic-Thermodynamic Promotion Engineering toward High-Density Hierarchical and Zn-Doping Activity-Enhancing ZnNiO@CF for High-Capacity Desalination
Source: Nanomicro Lett. 2024 Mar 4;16:143. doi: 10.1007/s40820-024-01371-y (PMC11329485; doi:10.1007/s40820-024-01371-y)
Supplement: Supplementary file 1 — Supplementary file1 (PDF 1034 kb) [file 40820_2024_1371_MOESM1_ESM.pdf]

Supporting Information for

Kinetic-Thermodynamic Promotion Engineering toward High-Density Hierarchical and Zn-Doping Activity-Enhancing ZnNiO@CF for High-Capacity Desalination

Jie Ma[1,2,3], Siyang Xing[2,3,5], Yabo Wang[2], Jinhu Yang[4], Fei Yu[1,*]

[1] College of Marine Ecology and Environment, Shanghai Ocean University, Shanghai 201306, P. R. China

[2] School of Civil Engineering, Kashi University, Kashi 844000, P. R. China

[3] Research Center for Environmental Functional Materials, College of Environmental Science and Engineering, Tongji University, 1239 Siping Road, Shanghai 200092, P. R. China

[4] School of Chemical Science and Engineering, Tongji University, 1239 Siping Road, Shanghai, 200092, P. R. China

[5] Department of Energy, Environmental & Chemical Engineering, Washington University in St. Louis, St. Louis, Missouri 63130, United States

*Corresponding author. E-mail: fyu@vip.163.com (Fei Yu)

Section S1 Materials Preparation

All reagents and chemicals were purchased from Sinopharm Chemical Reagent Co., Ltd., in chemical purity and used without further purification. All solutions were prepared using deionized water.

The preparation of the prefabricated carbon felt was based on the production method published by Liu et al. [S1] A piece of 3 cm × 3 cm × 0.1 cm carbon felt was immersed in a mixture of H_2SO_4 (98%) and HNO_3 (68%) at a volume ratio of 1:3 and placed in a water bath at 80 °C for 3 h. The carbon felt was then removed from the mixture and rinsed with deionized water to a neutral pH. Then the carbon felt was placed in absolute ethanol and sonicated for 30 min for ultrasonic cleaning. Subsequently, the carbon felt was washed with deionized water, placed in a vacuum drying oven, and dried at 60 °C for 24 h to prepare a prefabricated carbon felt electrode (pCF).

To prepare $Zn_xNi_{1-x}(OH)_2$ carbon felt electrode, 6 g of urea, 3.7 g of NH_4F, 14.75 g of $Ni(NO_3)_2·6H_2O$, and 1.48 g of $Zn(NO_3)_2·6H_2O$ were dissolved in a volume of 500 mL of deionized water at room temperature in to obtain mixture A. The pH of the pre-solution was adjusted to 4 by adding HNO_3 and NaOH. Then, the pCF was placed in a Teflon-lined stainless steel hydration reactor, and pre-solution A was added to immerse the pCF. The hydration reactor was sealed at a constant temperature (140 °C) for 2 h. After naturally being cooled to room temperature, the carbon felt was gently washed

with deionized water, and dried in a 60 °C vacuum drying oven for 24 h to obtain a prefabricated carbon felt electrodes with surface loading of $Zn_xNi_{1-x}(OH)_2$.

The dried $Zn_xNi_{1-x}(OH)_2$ was placed in a tube furnace, and nitrogen gas was introduced at a rate of 50 mL min^{-1} and heated to 500 °C at a heating rate of 5 $°C·min^{-1}$ for 2 h. This process was followed by annealing the nitrogen for 4 h to normal temperature to obtain a $Zn_xNi_{1-x}O$ electrode. Then, the mass of the loaded material in a single piece is obtained by weighing the mass of the sample and subtracting the mass of the dried prefabricated CFs.

To examine the effect of basicity in a hydrothermal environment, the same procedure was repeated with varied pH value (2, 4 and 6) of the pre-solution and the electrodes were named $Zn_{0.2}Ni_{0.8}O$-2, $Zn_{0.2}Ni_{0.8}O$-4 and $Zn_{0.2}Ni_{0.8}O$-6, respectively. In the section 2.2-2.5 parts, we mainly focus on the influence of Zn doping and we choose pH = 4 as the reaction condition, so we just set names of samples as $Zn_xNi_{1-x}O$. Furthermore, to probe the role of Zn, the same procedure was repeated with different Zn contents (0.59, 1.19, and 2.36 g) such that the molar ratio of Zn to Ni was 0.1, 0.2 and 0.4, respectively; these samples were denoted $Zn_{0.1}Ni_{0.9}O$, $Zn_{0.2}Ni_{0.8}O$ and $Zn_{0.4}Ni_{0.6}O$ respectively.

Section S2 Characterizations

The surface morphology was characterized by scanning electron microscopy (SEM, Hitachi S-4800/EX-350, Japan). The crystal structure was analyzed using X-ray diffraction (XRD D8 ADVANCE, Bruker AXS, Germany) operated at 40 mA and 45 kV with Cu Kα radiation (λ = 0.154 nm; 5°/min). X-ray photoelectron spectroscopy (XPS) analysis was carried out in a ThermoFisher ESCALAB 250Xi spectrometer using monochromated Al Ka X-rays (hν = 1486.6 eV) with a base pressure of 1×10^{-9} Torr. Survey scans determined between 1100 and 0 eV revealed the overall elemental compositions of the sample, and regional scans for specific elements were performed. The peak energies were calibrated by placing the major C 1s peak at 284.8 eV. The wettability was reflected by the water contact angle (POWEREACH-JC2000D2W). The specific surface area and pore size distribution were calculated from the adsorption/desorption isotherms of N_2 at 77 K by the multipoint BET, BJH, and DFT method using a BELSORP Max instrument (BEL), and the sample was degassed at 373 K for 4 h prior to the measurements. The hydrophilicity of the samples was studied by the water contact angle (WCA) test using an optical contact angle measuring device (POWEREACH JC2000, China).

Section S3 Electrochemical Test

The $Zn_xNi_{1-x}O$ were directly used as working electrode. The area of the working electrode used in the electrochemical tests was 1 cm ×1 cm. An electrochemical station (CHI660D, Chenhua Instruments Co.) was used for all electrochemical tests. CV was swept between -0.4 and 0.8 V under certain scan rates (1-60 mV s^{-1}), and GCD was measured at the uniform voltage window with various specific currents (1 -

6 mA cm^{-2}). EIS spectra were tested at the frequency range of 10^5 Hz to 10^{-2} Hz with an amplitude of 5 mV. Cyclic voltammetry (CV), galvanostatic charging/discharging (GCD), and electrochemical impedance spectroscopy (EIS) tests were conducted on a three-electrode system consisting of a working electrode, Pt (counter electrode), and Ag/AgCl (reference electrode) in 1 M NaCl.

A kinetic analysis can investigate the charge storage and ion removal kinetics. Specifically, one can derive key information via analysis of the dependency of the measured current with the sweep rate [S2], stated in Eqs. (S1) and (S2):

$$i = av^b \tag{S1}$$

$$\log(i) = blog(v) + \log(a) \tag{S2}$$

The b-value can be determined from the plot's slope between log (i) and log (v) and indicates the charge storage mechanism.

It is also possible to quantify the percentage of surface-controlled capacitance (capacitor-like contribution) corresponding with either a perfect diffusion-limited system (b-value of 0.5) or a perfect capacitor (b-value of 1.0). The closer it is to one, the more perfect is the pseudocapacitive response. A beneficial calculation for this consideration is the use of Eq. (S3) which is often referred to as Dunn analysis:

$$i(V) = k_1 + k_2 v^{1/2} \tag{S3}$$

Section S4 Desalination Experiments

The flow-by CDI stack is composed of glass plates, silica gel gaskets, a cation/anion exchange membrane (CEM/AEM), and a chamber with dimensions of 0.8 cm × 4 cm × 4 cm. An activated carbon electrode served as the anode, and a $Zn_xNi_{1-x}O$ electrode served as the cathode with the area of 3 cm × 3 cm. The batch mode was used in this experiments, and real-time conductivity was monitored by a conductivity meter (METTLER TOLEDO S230). Deionized water was used to cleanse the apparatus without applying voltage for 24 h, and the NaCl solution was then pumped into the CDI cell and returned to the feed container during the CDI process until the conductivity remained steady. All desalination experiments were carried out under a constant current with a fixed flow rate (30 mL min^{-1}), a fixed initial NaCl concentration (1000 mg L^{-1}) and a fixed volume (40 mL) at room temperature (295 K), while other operational parameters, including the different activated electrodes ($Zn_xNi_{1-x}O$), current density (600, 1000, 1500, 2000, 2500 and 3000 mA cm^{-2}), and cutoff voltage (0.6, 0.8, 1.0, 1.2, 1.4, 1.6 or 1.8 V), were varied to obtain the best desalination capacity. The NaCl concentration (milligrams per liter) was derived from the standard curve of conductivity and concentration. The removal capacity of NaCl based on the mass of $Zn_xNi_{1-x}O$ were calculated as shown below:

$$desalination\ capacity(Q) = \frac{(C_0 - C_e)V}{M}\ (\text{mg of NaCl/g of Zn}_x\text{Ni}_{1-x}\text{O}) \tag{S4}$$

and specific energy consumption (SEC, kWh kg^{-1} NaCl)

$$E_{in} = \int_{\Delta t_{cycle}} iv dt \quad where\ iv > 0 \tag{S5}$$

$$E_{out} = \int_{\Delta t_{cycle}} iv dt \quad where\ iv < 0 \tag{S6}$$

$$E_v = \frac{E_{in} - \eta E_{out}}{V_d} \tag{S7}$$

$$SEC = \frac{E_{in} - \eta E_{out}}{3.6(C_0 - C_e)V_d} \text{ (kWh/kg of NaCl)} \quad S(8)$$

where C_0 and C_e are the NaCl concentration (mg L^{-1}) at initial and equilibrium stages, respectively, M is the mass of $Zn_xNi_{1-x}O$ loading on one piece of pCF, V_d is the total NaCl volume (L), A is the area of the electrode (cm^2), v represents the voltage window (V), i is the current (mA), and t is the duration of one desalination cycle. and η is the fraction of E_{out} actually recovered and reused to power another charging phase. Theoretically, the η approximately equals to 1.

Section S5 EQCM-D Measurements

EQCM (QSense Explorer Electrochemistry, Biolin Scientific, Sweden) was used to carried out the measurement. The slurry (containing 80 wt% $Zn_{0.2}Ni_{0.8}O$, 10 wt% carbon black and 10 wt% PVDF in NMP) was coated onto quartz-crystal surface with Au electrode (Biolin Scientific, Sweden) with 0.79 cm^2 electrochemically active surface area. The quartz crystal sensor followed by vacuum drying dried at 60 °C for 3 h. In QCM electrochemical module, RE was Ag/AgCl, CE was a Pt mesh and the $Zn_{0.2}NiO$ electrode coated quartz crystal sensor was WE. The electrolyte was NaCl (10 mmol L^{-1}). QSoft401 software was used to gather the frequency (Δf, Hz) and dissipation factor changes (ΔD). The calculation of mass change (Δm, g) was estimated according to the Sauerbrey equation (Eq. S9), and then the mpe (mass accumulated per mole of electron transferred) values were estimated according to the Sauerbrey equation(Eq. S9) and Faraday's law (Eq. S10) [S3]:

$$\Delta m = -C_f \frac{\Delta f}{n} = -\frac{A(\mu_q \rho_q)^{0.5} \Delta f}{2 f_0^2} \tag{S9}$$

$$mpe = nF \cdot \frac{\Delta m}{Q} \tag{S10}$$

Where C_f is the mass sensitivity constant (17.7 ng cm^{-1} Hz^{-1} for 5 MHz crystals), n is the overtone number (1,3…n), Δm is the mass change, Δf is the change in resonance frequency, f_0 is the fundamental resonance frequency (7.995 MHz), A is the surface area of the electrode, F (C mol^{-1}) is the Faraday constant (96,485), μ_q is the shear modulus of quartz (2.947 × 10^{11} g cm^{-1} s^{-2}), ρ_q is the density of quartz (2.684 g cm^{-3}).

The charge capacity ΔQ(C) was calculated by Eq. S11:

$$\Delta Q = \int I \times dt \tag{S11}$$

Where I (A) is the charge current, t (s) is the time. ΔQ is set to 0 when ΔQ accumulates the most.

Section S6 Theoretical Calculation

We used the DFT as implemented in the Vienna Ab initio simulation package (VASP) in all calculations. The exchange-correlation potential is described by using the generalized gradient approximation of Perdew-Burke-Ernzerhof (GGA-PBE). The projector augmented-wave (PAW) method is employed to treat interactions between ion cores and valence electrons. The plane-wave cutoff energy was fixed to 500 eV. Given structural models were relaxed until the Hellmann–Feynman forces smaller than -0.02 eV/Å and the change in energy smaller than 10^{-5} eV was attained. During the relaxation, the Brillouin zone was represented by a Γ centered k-point grid of 2×2×1. A vacuum layer of around 20 Å was added in the direction perpendicular to the surface to eliminate the spurious interlayer interaction.

Supplementary Figures and Tables

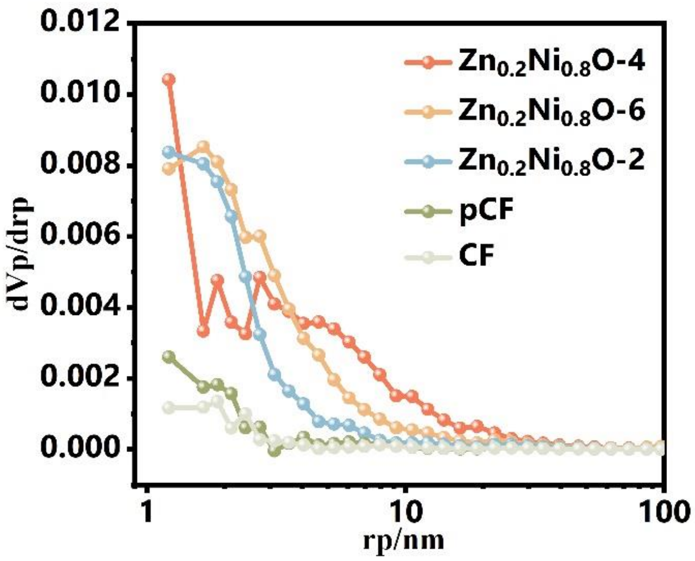

Fig. S1 BJH pore size analysis of CF, pCF, $Zn_{0.2}Ni_{0.8}O$-2, $Zn_{0.2}Ni_{0.8}O$-4 and $Zn_{0.2}Ni_{0.8}O$-6 electrodes

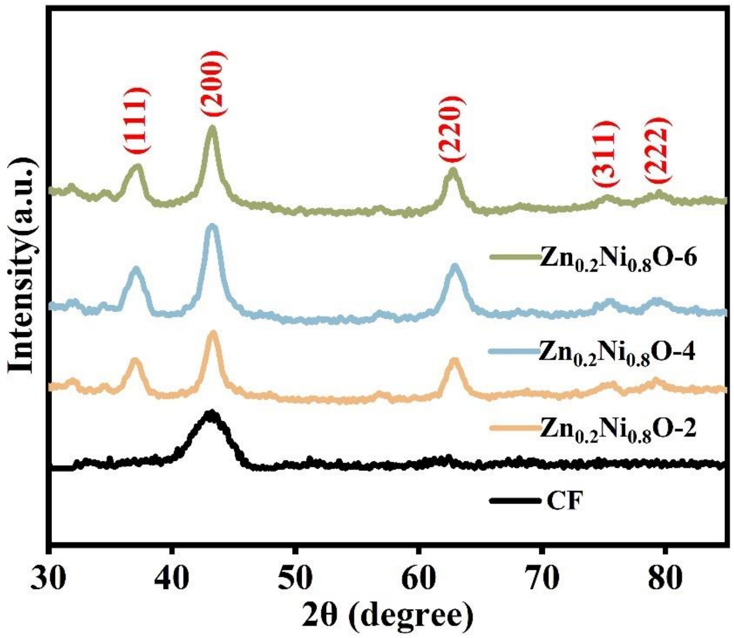

Fig. S2 X-ray diffraction patterns of $Zn_{0.2}Ni_{0.8}O$ with different pH values

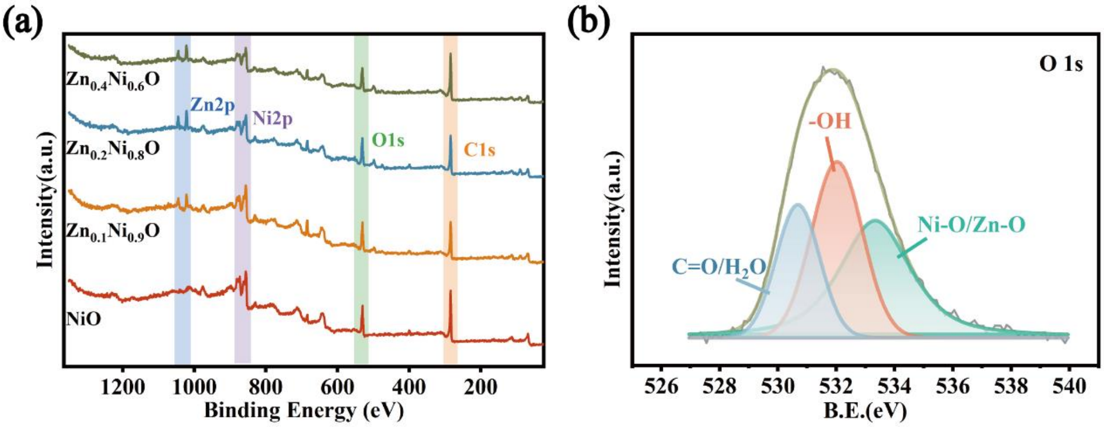

Fig. S3 XPS survey scan of $Zn_xNi_{1-x}O$ **(a)** and O1s spectra of $Zn_xNi_{1-x}O$ **(b)**

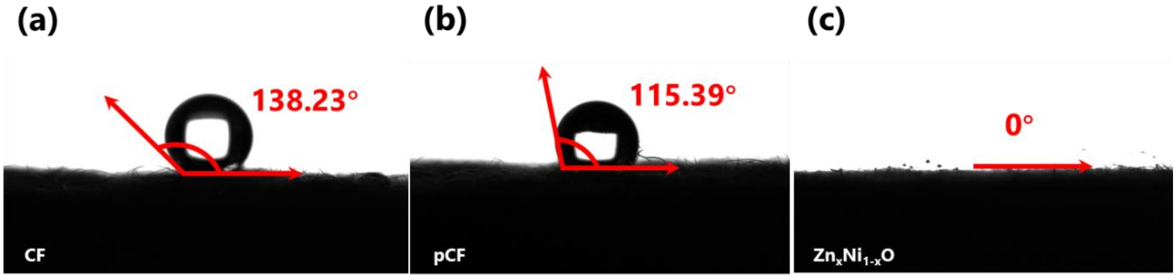

Fig. S4 The optical image of water contact angle on the surfaces of CF **(a),** pCF **(b),** and $Zn_xNi_{1-x}O$ electrodes **(c)**

The water contact angle was measured to determine the hydrophilicity of the prepared samples. The water contact angles of CF and pCF are 138.23°, 115.39°, and all the $Zn_{0.2}Ni_{0.8}O$ simples of different pH are 0° (**Fig. S1**). The CF and pCF electrodes

have a higher water contact angle and poor hydrophilicity; while the water droplets instantly soak into the surface of the $Zn_{0.2}Ni_{0.8}O$ electrode, indicating that the $Zn_{0.2}Ni_{0.8}O$ has excellent surface wettability. The excellent hydrophilicity facilitates the migration and penetration of ions in the aqueous solution and thus has a positive impact on the performance of CDI.

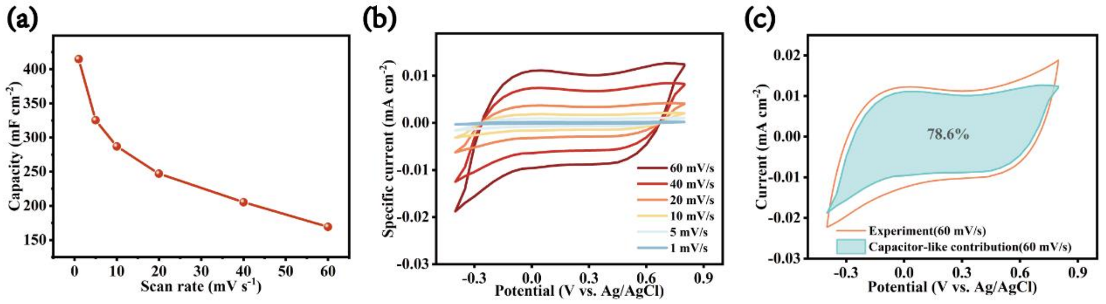

Fig. S5 (a) Specific capacitance (F cm^{-2}) of $Zn_{0.2}Ni_{0.8}O$ with respect to scan rate (mV/s). **(b)** The curves of pseudocapacitive contribution of the cyclic voltammogram at different scan rates. **(c)** Estimation of pseudocapacitive contribution of the cyclic voltammogram at 60 $mV{\cdot}s^{-1}$

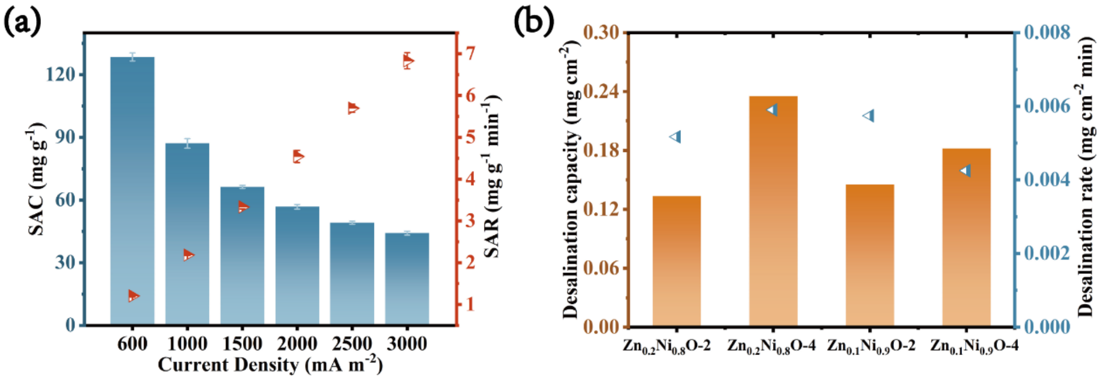

Fig. S6 (a) SAC and SAR of $Zn_{0.2}Ni_{0.8}O$ at different current density. **(b)** SAC and SAR of various electrodes at 1000 mA m^{-2}

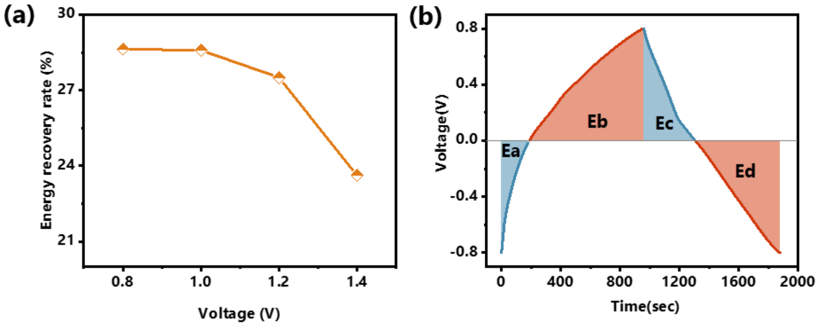

Fig. S7 (a) energy recovery rate of different voltage, **(b)** Schematic diagram of energy recovery (*E*a and *E*c represent theoretically recyclable energy; *E*b and *E*d represent consumed energy)

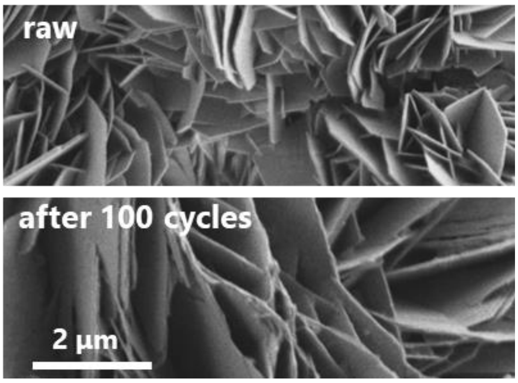

Fig. S8 SEM images of the $Zn_{0.2}Ni_{0.8}O$ electrode before and after a long-cycle desalination experiment

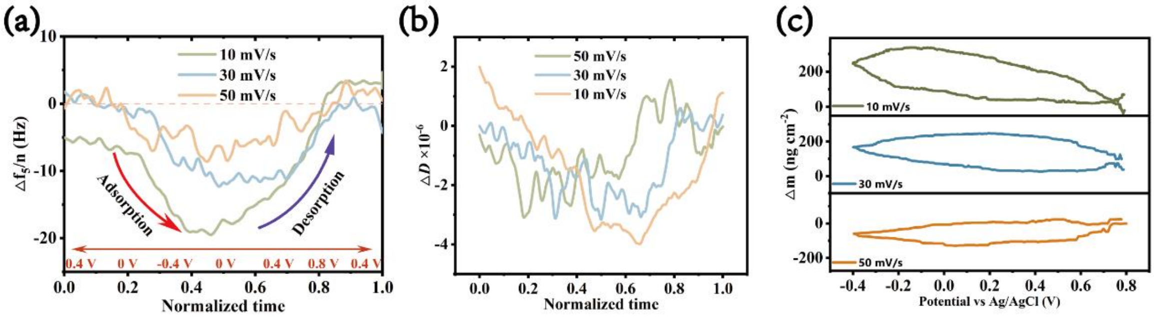

Fig. S9 Time-dependent changes in $f_5/5$ **(a)** and D_5 **(b)** of $Zn_{0.2}Ni_{0.8}O$ at different sweep rates during the 3rd CV cycle measured via EQCM. **(c)** Mass change of $Zn_{0.2}Ni_{0.8}O$ electrode during the CV cycles in different scan rate

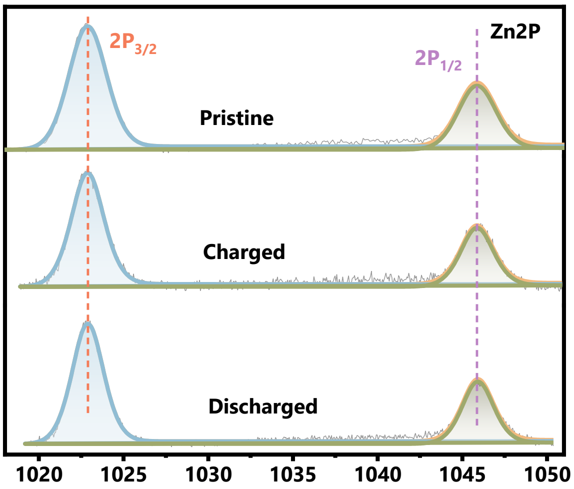

Fig. S10 Ex-situ XPS spectra/data of Zn 2p $Zn_{0.2}Ni_{0.8}O$ electrodes in pristine states and $Zn_{0.2}Ni_{0.8}O$ electrodes in fully adsorption/desorption states after the third desalination cycle at 1000 mA cm^{-2}

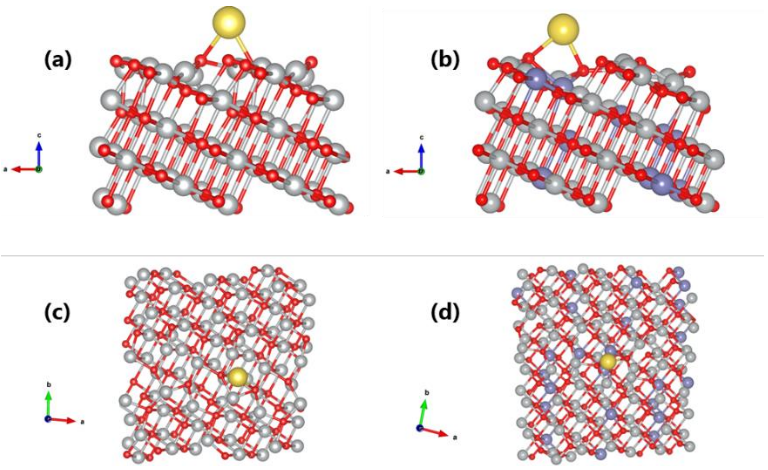

Fig. S11 Side view **(a, b)** and top view **(c, d)** of relaxed adsorption configurations for corresponding E_{ads} for Na^+ on NiO and $Zn_{0.2}Ni_{0.8}O$ with (225) surfaces

Table S1 Surface area, pore size, and pore volume of different $Zn_xNi_{1-x}O$ carbon felt electrodes

Electrodes	BET surface area (m^2 g^{-1})	Pore volume (cm^3 g^{-1})	Pore size(nm)
CF	2.4336	0.00362	5.9555
pCF	9.4108	0.00799	3.3957
$Zn_{0.2}Ni_{0.8}O$-2	21.849	0.02463	4.5084
$Zn_{0.2}Ni_{0.8}O$-4	94.341	0.07954	3.3723
$Zn_{0.2}Ni_{0.8}O$-6	42.984	0.04664	4.3405

Table S2 R_{int} and R_{ct} of different $Zn_xNi_{1-x}O$ carbon felt electrodes

Electrodes	R_{int} (Ω)	R_{ct} (Ω)
NiO	5.46	2.327
$Zn_{0.1}Ni_{0.9}O$	4.671	4.188
$Zn_{0.2}Ni_{0.8}O$	5.096	0.44
$Zn_{0.4}Ni_{0.6}O$	5.94	2.592

Table S3 Comparison of SAC of various CDI electrodes

Electrode	Applied Voltage/Specific Current	Salinity (mg L^{-1})	SAC (mg g^{-1})	SAR (mg g^{-1} min^{-1})	Energy Consumption (kWh kg^{-1})	References
$Zn_{0.2}Ni_{0.8}O$	~22.2 mA g^{-1}	1000	128.9	1.21	0.164	This work
CNF-4	1.2 v	800	63.51	4.38	1.07	[S4]
HCl-NC	1.2 V	1000	100.3	~1.75	0.394	[S5]
Electrospun PCF	1.0 V	500	30.4	~3	0.366	[S6]
PPy-p-TS//PPy-ClO_4	0.75 V	585	74.3	2.46	0.288	[S7]

PB/PANI	100 mA g^{-1}	500	133.3	1.8	~1.01	[S8]
NVP/C	50 mA g^{-1}	5850	137.2	4.56	0.463	[S9]
NVOPF/rGO	50 mA g^{-1}	1000	~115	~2.24	0.35	[S10]
Porous $Ti_3C_2T_x$	1.2 V	10000	118	-	-	[S11]
mPDA/MXene	1.5 V	1000	37.72	1.27	0.69	[S12]
N-$Ti_3C_2T_x$	1.2 V	5000	43.5	-	-	[S13]
Functionaltion Mxene	1.2 V	5000	49	2.92	0.38	[S14]
$W_{18}O_{49}/Ti_3C_2$	1.2 V	500	29.25	0.97	0.564	[S15]
TiO_2/Ti_3C_2	15 mA g^{-1}	500	75.62	0.68	0.49	[S16]

Table S4 The peak positions/binding energy difference of peaks and the contribution ratio of the fitted chemical species of Ni 2$p_{3/2}$

**Ni 2p3/2**	**Peak/eV**			**Ratio/%**	
	**NiO**	**$Zn_{0.2}Ni_{0.8}O$**		**NiO**	**$Zn_{0.2}Ni_{0.8}O$**
**Pristine**	857.00	856.75	2+	46.3	43.6
			3+	53.7	56.4
**Charged**	856.10	855.28	2+	58.7	65.0
	△E=0.9eV	△E=1.47eV	3+	41.3	35.0
**Discharged**	856.29	855.18	2+	52.5	57.0
	△E=0.71eV	△E=1.57eV	3+	47.5	43.0

Supplementary References

[S1] S. Liu, R. Wang, C. Ma, D. Yang, D. Li et al., Improvement of electrochemical performance via enhanced reactive oxygen species adsorption at ZnO–NiO@rGO carbon felt cathodes in photosynthetic algal microbial fuel cells. Chem. Eng. J. **391**, 123627 (2020). https://doi.org/https://doi.org/10.1016/j.cej.2019.123627

[S2] J. Wang, J. Polleux, J. Lim, B. Dunn, Pseudocapacitive contributions to electrochemical energy storage in TiO_2 (anatase) nanoparticles. J. Phys. Chem. C **111**(40), 14925-14931 (2007). https://doi.org/10.1021/jp074464w

[S3] Z.-W. Yin, X.-X. Peng, J.-T. Li, C.-H. Shen, Y.-P. Deng et al., Revealing of the activation pathway and cathode electrolyte interphase evolution of Li-rich $0.5Li_2MnO_3{\cdot}0.5LiNi_{0.3}Co_{0.3}Mn_{0.4}O_2$ cathode by in situ electrochemical quartz crystal microbalance. ACS Appl. Mater. Inter. **11**(17), 16214-16222 (2019). https://doi.org/10.1021/acsami.9b02236

[S4] R. Liu, Y. Wang, Y. Wu, X. Ye, W. Cai, Controllable synthesis of nickel–cobalt-doped Prussian blue analogs for capacitive desalination. Electrochim. Acta. 141815 (2023). https://doi.org/https://doi.org/10.1016/j.electacta.2023.141815

[S5] M. Liang, N. Liu, X. Zhang, Y. Xiao, J. Yang et al., A reverse-defect-engineering

strategy toward high edge‐nitrogen‐doped nanotube‐like carbon for high-capacity and stable sodium ion capture. Adv. Funct. Mater. (2022). https://doi.org/10.1002/adfm.202209741

[S6] T. Liu, J. Serrano, J. Elliott, X. Yang, W. Cathcart et al., Exceptional capacitive deionization rate and capacity by block copolymer–based porous carbon fibers. Sci. Adv. **6**(16), eaaz0906 (2020). https://doi.org/10.1126/sciadv.aaz0906

[S7] H.-Y. Huang, Y.-H. Tu, Y.-H. Yang, Y.-T. Lu, C.-C. Hu, Dopant-designed conducting polymers for constructing a high-performance, electrochemical deionization system achieving low energy consumption and long cycle life. Chem. Eng. J. **457**, 141373 (2023). https://doi.org/https://doi.org/10.1016/j.cej.2023.141373

[S8] W. Shi, X. Liu, T. Deng, S. Huang, M. Ding et al., Enabling superior sodium capture for efficient water desalination by a tubular polyaniline decorated with prussian blue nanocrystals. Adv. Mater. **32**(33), 1907404 (2020). https://doi.org/https://doi.org/10.1002/adma.201907404

[S9] J. Cao, Y. Wang, L. Wang, F. Yu, J. Ma, $Na_3V_2(PO_4)_3$@C as faradaic electrodes in capacitive deionization for high-performance desalination. Nano Lett. **19**(2), 823-828 (2019). https://doi.org/10.1021/acs.nanolett.8b04006

[S10] S. Xing, Y. Cheng, F. Yu, J. Ma, $Na_3(VO)_2(PO_4)_2F$ nanocuboids/graphene hybrid materials as faradic electrode for extra-high desalination capacity. J. Colloid Interface Sci. **598**, 511-518 (2021). https://doi.org/10.1016/j.jcis.2021.04.051

[S11] W. Bao, X. Tang, X. Guo, S. Choi, C. Wang et al., Porous cryo-dried MXene for efficient capacitive deionization. Joule **2**(4), 778-787 (2018). https://doi.org/https://doi.org/10.1016/j.joule.2018.02.018

[S12] Q. Li, X. Xu, J. Guo, J. P. Hill, H. Xu et al., Two-dimensional MXene-polymer heterostructure with ordered in‐plane mesochannels for high‐performance capacitive deionization. Angew. Chem. Int. Ed. (2021). https://doi.org/10.1002/anie.202111823

[S13] A. Amiri, Y. Chen, C. Bee Teng, M. Naraghi, Porous nitrogen-doped MXene-based electrodes for capacitive deionization. Energy Stor. Mater. **25**, 731-739 (2020). https://doi.org/https://doi.org/10.1016/j.ensm.2019.09.013

[S14] Z. Bo, Z. Huang, C. Xu, Y. Chen, E. Wu et al., Anion-kinetics-selective graphene anode and cation-energy-selective MXene cathode for high-performance capacitive deionization. Energy Stor. Mater. **50**, 395-406 (2022). https://doi.org/https://doi.org/10.1016/j.ensm.2022.05.042

[S15] J. Liang, J. Yu, W. Xing, W. Tang, N. Tang et al., 3D interconnected network architectures assembled from $W_{18}O_{49}$ and Ti_3C_2 MXene with excellent

electrochemical properties and CDI performance Chem. Eng. J. **435**, 134922 (2022). https://doi.org/https://doi.org/10.1016/j.cej.2022.134922

[S16] N. Liu, L. Yu, B. Liu, F. Yu, L. Li et al., Ti_3C_2-MXene partially derived hierarchical 1D/2D TiO_2/ Ti_3C_2 heterostructure electrode for high-performance capacitive deionization. Adv. Sci. **10**(2), e2204041 (2023). https://doi.org/10.1002/advs.202204041
